# A Combination of Chemometrics and Quantum Mechanics Methods Applied to Analysis of Femtosecond Transient Absorption Spectrum of Ortho-Nitroaniline

**DOI:** 10.1038/srep19364

**Published:** 2016-01-19

**Authors:** Jing Yi, Ying Xiong, Kemei Cheng, Menglong Li, Genbai Chu, Xuemei Pu, Tao Xu

**Affiliations:** 1 College of Chemistry, Sichuan University, Chengdu 610064, People’s Republic of China; 2 Institute of Chemical Material, China Academy of Engineering Physics (CAEP), Mianyang 621900, People’s Republic of China; 3 Science and Technology on Plasma Physics Laboratory, Research Center of Laser Fusion, CAEP, Mianyang 621900, People’s Republic of China

## Abstract

A combination of the advanced chemometrics method with quantum mechanics calculation was for the first time applied to explore a facile yet efficient analysis strategy to thoroughly resolve femtosecond transient absorption spectroscopy of ortho-nitroaniline (ONA), served as a model compound of important nitroaromatics and explosives. The result revealed that the ONA molecule is primarily excited to S3 excited state from the ground state and then ultrafast relaxes to S2 state. The internal conversion from S2 to S1 occurs within 0.9 ps. One intermediate state S* was identified in the intersystem crossing (ISC) process, which is different from the specific upper triplet receiver state proposed in some other nitroaromatics systems. The S1 state decays to the S* one within 6.4 ps and then intersystem crossing to the lowest triplet state within 19.6 ps. T1 was estimated to have a lifetime up to 2 ns. The relatively long S* state and very long-lived T1 one should play a vital role as precursors to various nitroaromatic and explosive photoproducts.

The transient absorption spectroscopy is a pump-probe time-resolved optical spectroscopy technique, in which the “pump” pulse triggers a photo-induced chemical process and the subsequent system evolution is monitored by a delayed “probe” pulse. In order to investigate fast processes, very short pulse is necessary. So far, dramatic advances in optical pulse technology have enable pulses shorter than 100 fs available, advancing development of femtosecond transient absorption spectroscopy (femto-TA)^1^,^2^,^3^ Since femtosecond pulses are shorter than the time scale for most intramolecular motions, they enables to measure ultrafast photoexcited processes occurring in the excited electronic states, which play a vital role in controlling the use and performance of the photochemical and photobiological systems^2^,^4^. Many chemical compounds and biological molecules have been investigated by the technique in order to reveal their photodynamic properties^5^,^6^,^7^,^8^,^9^,^10^. These works already revealed that the photodynamics behavior after photoexcitation is quite complex, in which more than one transient species generally exist. As known, the transient absorption spectroscopy determines the change of optical density (∆OD) through the pump-probe technique, which contains overlapping signals of the excited-state absorption (ESA), stimulated emission (SE) and ground state bleach (GSB) arising from the multi-species in the excited state at the different delay-time scale^11^. Due to the limitation of purely experimental technique in characterizing the excited states and differentiating the overlapping spectrum, it has emerged as an important task to unravel the characteristic time profiles of the different components contributed to the overlapping signals by means of introducing some other advanced techniques to assist the resolving of femto-TA. Chemometrics^12^, involved in mathematical and statistical models, has been applied in spectroscopy for more than 40 years, multivariate calibration methods of which play an important role in multicomponent resolution and quantification^13^,^14^,^15^. In particular, multivariate curve resolution-alternating least squares MCR–ALS^16^,^17^ is the most widely used method for the analysis of unknown spectroscopic mixtures in both time and spectral direction, which provides a powerful alternative way to complement the resolving of femto-TA data^18^,^19^. However, it is still limited for purely applying the chemometrics method to thoroughly map the photodynamic mechanism of the excited states since the method cannot characterize the structures and the transition nature of excited states, which are associated with the electronic level of the molecules. As well accepted, quantum mechanics (QM) calculation is a mature technique to study molecular properties at the electronic level^20^, including molecular structures, chemical reactions, spectral properties, and so on. Consequently, QM method has been successfully applied to study the structures and the transition natures of the excited states in nanosecond^21^,^22^ and femtosecond TA spectra so as to explore the decay mechanism after the excitation^4^,^23^,^24^,^25^,^26^. These previous works clearly exhibit that the chemometrics and QM methods have individual advantages in resolving the femto-TA spectrum. The chemometrics method could decompose the overlapping spectra into spectrum of pure species by means of a mathematic separation while the QM method could characterize the structure and transition nature of the excited state. Thereby, we can assume that a combination of the advantages from the two techniques should provide a more efficient and in-depth way for resolving the femto-TA spectrum. However, to the best of our knowledge, the joint method has not been proposed in previous works, which inspire our research interests. Namely, we hope to explore an effective strategy in globally resolving femto-TA data absorption spectroscopy by means of a combination of the QM and chemometrics methods.

In the work, we choose ortho-nitroaniline (ONA) as a case study, mainly based on the following two reasons. First, the interest in the ONA stems from the background of explosives with multi-nitro groups and amino groups. As revealed, the excited electronic state decay can lead to local heating effect called “hot spot” of energy materials in the energy conversion, which plays an important role in detonation course and impact sensitivity of explosives^26^,^27^. However, there are very limited studies on the decay of the electronically excited states of the nitro- and amino- aromatic explosives since the process is ultrafast and exothermic, involving in the safety. Only recently, with the aid of a simple singular-value decomposition (SVD) method, Chu^28^ used femto-second transient absorption to study the relaxation dynamics behavior of 2, 2′, 4, 4′, 6, 6′-hexanitrostilbene (HNS) after excitation at 266 nm and proposed a relaxation process of S_2_ → S^*^ → S_1_ → T_1_. Thereby, elucidation of the initial decay and the dynamics behavior of excited electronic states has been quietly absent and highly desirable for the explosives. Alternatively, there is also an effective way to access the dynamics behavior of the excited state for the applied explosives through studying their simple prototypes. The approach has been commonly adopted to assist studies on the structure and function of the explosives. For example, by means of nanosecond energy resolved and femtosecond time resolved spectroscopic techniques, E. R. Bernstein’s group^29^ studied some explosives model systems, such as nitromethane (NM), dimethylnitramine (DMNA) and isopropylnitrate (IPN) in order to obtain the initial decomposition of electronic excited states in the energetic materials. In fact, compared to the simple model systems above, ONA has more similar structure to the nitro aromatic explosives, such as 1,3,5-triamino-2,4,6-trinitrobenzene (TATB), 1,3-diamino-2,4,6-trinitro- benzene (DATB) and 1-amino-2,4,6-trinitrobenzene (TNA). The intramolecular hydrogen transfer of ONA, as a model system of poly-nitro aromatic explosives, was already studied by Zhang^30^ using infrared spectroscopic and quantum chemistry methods. However, the dynamics behavior of the excited states has not been addressed for ONA so far. Thus, the observations from the fetmo-TA of ONA will advance understanding of the dynamics behavior of the excited states for more complicated explosives.

The second reason of selecting the ONA comes from the fact that ONA is also a simple and typical nitroaromatic compounds while the photochemistry and photophysics of the nitroacromatic compounds have aroused significant attention because they are closely associated with mutagenic and carcinogenic environmental pollutants, the photodynamics of the excited states of which play an essential role in natural decay from the atmosphere through light-induced reactions^26^,^31^,^32^,^33^. As mentioned above, the transient absorption studies were already performed for some singly nitroaromatic compounds and nitro-polyaromatic ones^23^,^24^,^26^,^33^,^34^ including para nitroaniline (PNA)^35^,^36^, an isomer of ONA. These works mainly concerned the intersystem crossing (ISC) dynamics process with help of QM calculation and revealed that the nitroacromatic compounds have significantly different ISC behaviors, dependent on their structures, the substituent types and positions as well as environments. Thus, the observations from the ONA will further extend our understanding of the photophysics of the nitroaromatics.

Based on the consideration above, we, herein, study the femto-TA spectrum of ONA by pump–probe technique in a range 400–710 nm with 35 fs resolution after excitation at 266 nm. Compared to 400 nm excitation wavelength used in PNA system, we used shorter excitation wavelength for ONA in order to gain insight into the relaxation process in higher excited levels. The short excitation wavelength close to 266 nm were adopted in the femto-TA spectra of some compounds^4^,^37^,^38^,^39^,^40^, including HNS^28^. The MCR-ALS in combination with QM calculation is for the first time adopted to explore an efficient resolving method to obtain a comprehensive description of a complete decay evolution process of the electronic excited states of ONA, which will provide valuable information for developing facile yet efficient analysis method for resolving the transient absorption spectrum used in the other fields. In addition, some novel observations were obtained from the work, which would advance understanding of electronic excited state decay for the nitro-aromatic compounds and the explosives.

## Results and Discussion

In the following, we first used the steady state absorption and QM calculation to identify the electronic excited state populated by quick excitation at 266 nm. Then, SVD and the evolving factor analysis (EFA) were performed to estimate the number of significant contribution in the relaxation process of the excited state. Based on the number of significant contribution, MCR-ALS was used to decompose the time-resolved spectra into pure spectra of overlapping transient species and their corresponding time-dependent concentration. With aid of QM calculations, we interpret the dynamics profiles and the pure spectra of main excited states of ONA transient absorption.

### Analysis of steady state absorption assisted with quantum mechanics calculation

**Figure 1:**
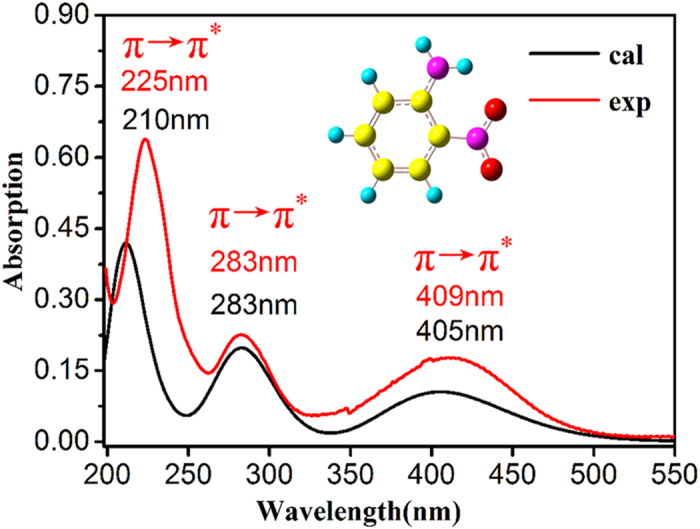
Steady-state absorption spectra and calculated absorption spectra of ONA in water with molecular structure of ONA as an inset.

Steady state absorption spectrum of ONA was measured in water and displayed in Fig. 1, which exhibits three wide intense absorption peaks at 225 nm, 283 nm and 409 nm. To gain insight into the transition nature of the absorption spectrum, we optimized ONA ground S_0_ structure in water at the level of B3LYP/6-311++ G (d, p) within the framework of polarizable continuum model (PCM). Based on the S_0_ optimized geometry, TD-DFT method was used to calculate the spectral properties of ONA in water at the same level. The calculated absorption spectrum is also depicted in Fig. 1 while Table 1 shows compilations of the excitation energies, oscillator strengths, transition character and dominant orbital contributions of the low-lying single excited states of ONA. As shown in Fig. 1, the calculated spectrum is very close to the experiment one, in which the three calculated absorption peaks at 210, 283 and 405 nm well reproduce the experiment ones at 225, 283 and 409 nm, respectively.

The good consistency between the calculated data and the experimental one confirms the reliability of the computational level adopted in the work. An inspection of Table 1 clearly shows that S_0_ → S_3_ transition has much stronger oscillator strength with f = 0.22 than the other transitions and its transition energy is calculated to be 4.38 eV, corresponding to the adsorption wavelength of 283 nm with π → π^*^ transition character. The calculated wavelength of 283 nm is close to the pumping wavelength at 266 nm used in the transient absorption spectrum. Taking together, it is convincing to confirm that the ONA molecular most probably be excited to the electronic excited S_3_ state after quick excitation at 266 nm.

### Conventional Analysis of Femtosecond transient absorption

**Figure 2:**
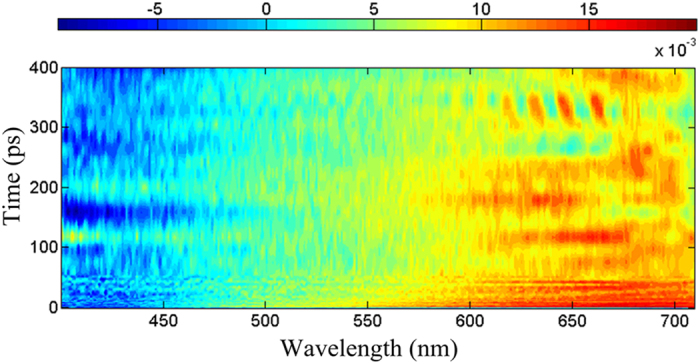
Two-dimensional transient absorption spectra of ONA in the range of 400–710 nm with time delay from 0 to 400 ps, measured in water.

Two-dimensional transient absorption spectrum of ONA was determined at various time delays after the 266 nm excitation pulse with 35 fs time resolution in water at room temperature. Figure 2 shows 2D plot of the spectrum, which covers the range from 400 nm to 710 nm and possess a long delay time of 400 ps. The negative signals (blue region) in the 400–460 nm range should correspond to the ground state bleaching (GSB) or stimulated emission (SE)^28^,^41^. The positive signals in the region of 460–710 nm should involve in excited state absorption mixed with the simulated emission from the high vibration excited states to the low ones. In addition, as shown in Fig. 2, the persistence absorption in the positive signal region is still strong without fading out even in a long delay time of 400 ps, implying an existence of long-lived triplet state absorption.

**Figure 3:**
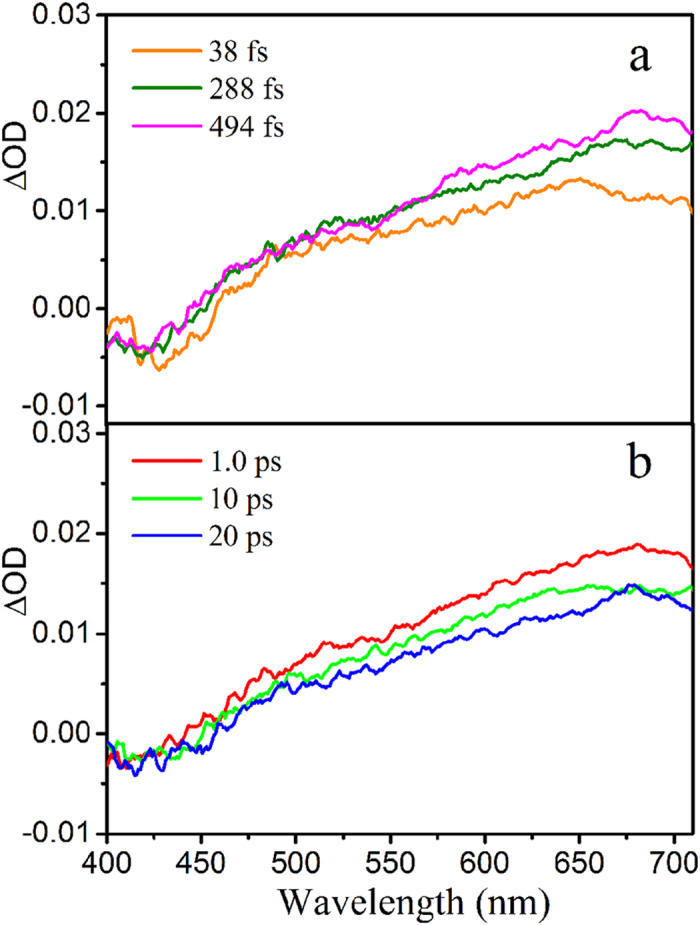
Some representative transient absorption spectra of ONA in water within the first 20 ps delay times.

Figure 3 further displays the representative transient absorption spectra within the first 20 ps decay time scale after 266 nm excitation. As can be seen from Fig. 3a, after quick excitation (viz., 38 fs), one relatively broad negative signal appeared at less than 460 nm region should be mainly ascribed to the transition in the electronic ground state absorption and partly mixed with stimulated emission from S_3_ excited state since the region almost corresponds to the band peaked at 409 nm of the steady state absorption (see Fig. 1). Within the time delay of 38–500 fs, the negative signal in the range of less than 420 nm gradually decay concomitant with the growth of the negative signal at 420–460 nm region, which should arise from the weakness of the ground state absorption and the increase of the stimulated emission. In addition, the broad positive absorption band between 460 nm and 710 nm gradually rises within first 500 fs (see Fig. 3a), implying appearance of some new excited states. After 500 fs, the positive absorption band gradually drop due to depopulation of the singlet electronic excited state. It is interesting to note that a sharp and positive peak appears near 675 nm at the 20 ps time delay (see Fig. 3b), implying a possibility of formation of one new excited state at 20 ps.

### MCR-ALS of Time-Resolved Spectra

**Figure 4:**
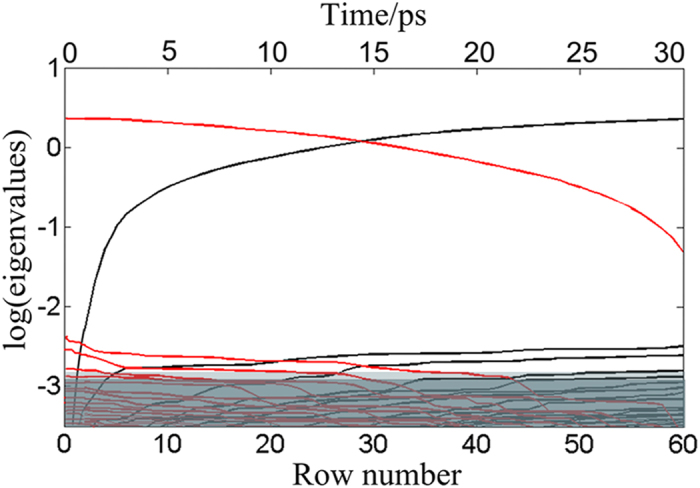
Evolving factor analysis (EFA) for transient absorption data of ONA; Black lines denote forward EFA while the red lines denote backward EFA. Eigenvalues (in log units) are plotted as a function of the row number of the data matrix and corresponding delay time.

In order to further identify the specific temporal evolution of the spectrum and dynamics behavior for the main excited states involved in the femtosecond transient absorption data, we used MCR-ALS method to decompose the transient absorption spectra of multiple components into pure spectra of the transient species and their time-dependent concentrations. As described in method part, EFA calculation was first performed to estimate the number of significant contributions for the S_3_ state relaxation process. To reduce computing time of EFA calculation, we only took the first 60 row into accounts, which corresponds to 30 ps. Figure 4 plots the principal component eigenvalues (in log units) obtained from the forward and backward analysis *versus* the delay time. A threshold separating major contributions from noise is set at log (eig) = −2.8, which leads to four significant contributions observed. As reflected by Fig. 4, in the forward direction, the first contribution and the second one emerges very quickly. The third and the fourth components appear a bit delayed. The analysis on the backward direction give similar result. Thereby, we could draw such a conclusion that there are four significant contributions in the relaxation process.

**Figure 5:**
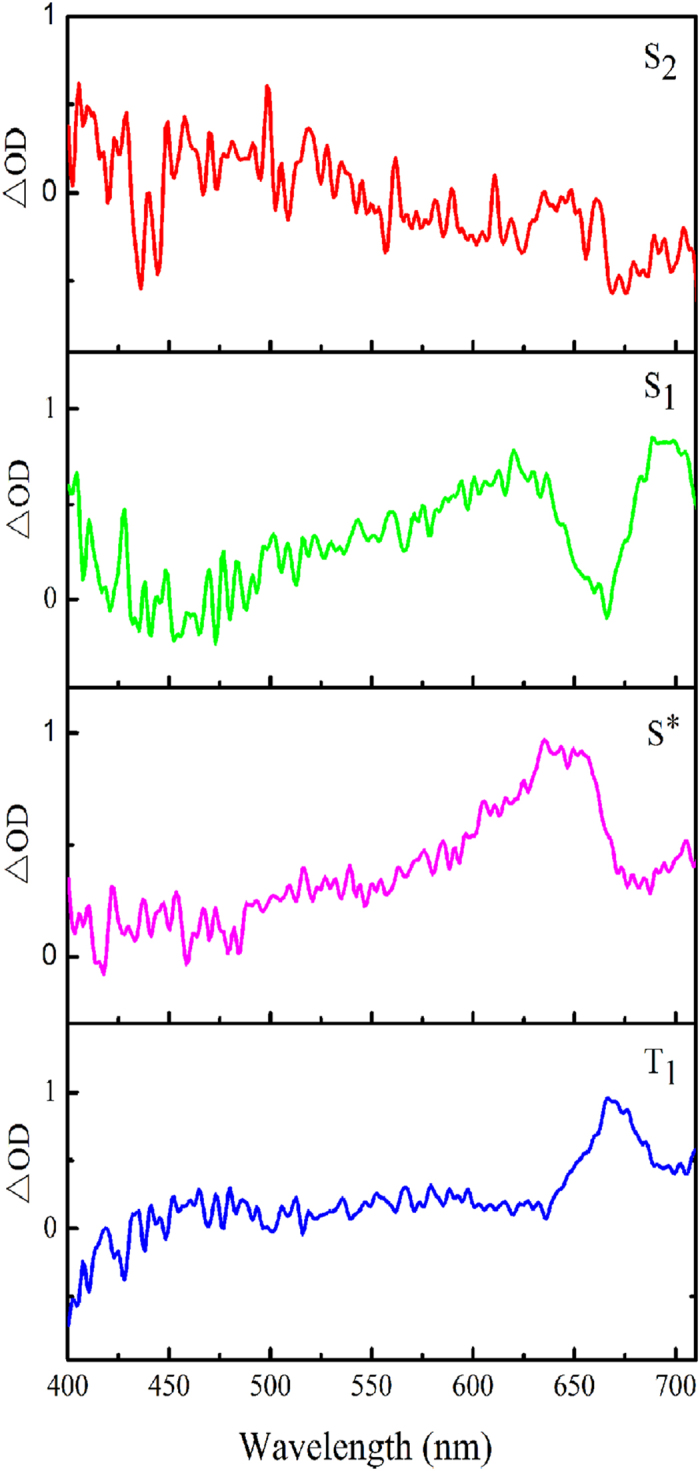
Spectroscopic evolution of the four main excited states of ONA identified by MCR-ALS analysis.

**Figure 6:**
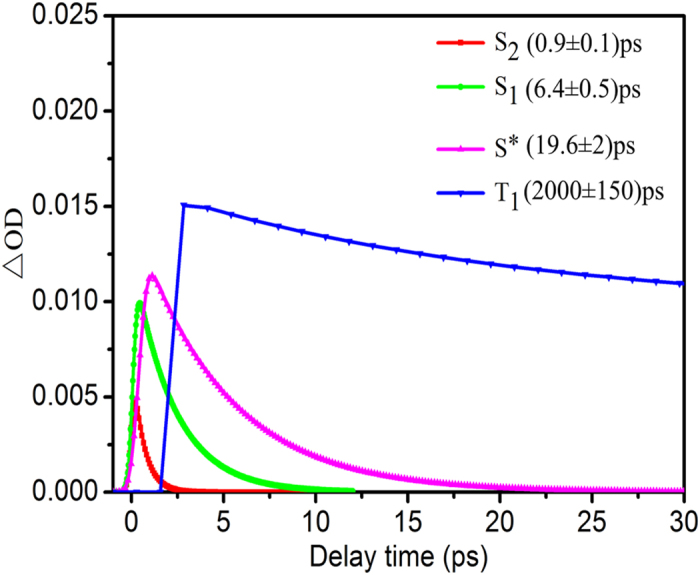
Time-dependent concentrations of the four main excited states of ONA, derived from the MCR-ALS and global fitting analysis. Their decay times and fitting errors are displayed in parentheses.

Based on the number of main excited states derived from the EFA analysis, the soft-modeling MCR-ALS analysis was applied to resolve the transient absorption spectra during 0–30 ps time range and obtain the initial estimates of time dependent concentration profiles ***C*** for sequential process. Figures 5 and 6 display the pure spectra of the four components (***S***^***T***^) and the corresponding time-dependent concentrations (***C***) obtained from the MCR-ALS analysis, respectively. A lack of fit was estimated to be 2.8% from the experimental matrix, indicating the reasonability of the MCR-ALS results.

### Assignment of the excited states significantly contributed to the Femtosecond transient absorption spectrum

#### S_2_ state

As revealed by QM calculation above, the ONA molecular most probably be excited to S_3_ state at 266 nm excitation. However, there were few experimental evidences for the S_3_ → S_2_ relaxation time, which may be resulted from the ultrafast internal conversion between the two singlet excited states. Only recently, theoretical observations from *ab initio* molecular dynamics simulation predicted that the internal conversion from S_3_ to S_2_ occur within 200 fs time scale for uracil^42^ and 25 fs time scale for DNA nucleobase cytosine^43^, providing a thereotical support for the ultrafast relaxation of S_3_ → S_2_. For ONA, the internal conversion from S_3_ state to S_2_ may be beyond the ~200 fs time resolution of the work. Consequently, we could not definitely identify the decay dynamics of S_3_ state from the fetmo-TA spectrum. Thereby, the spectrum of the first species was assigned to be S_2_ state. The corresponding time-dependent concentration gives a lifetime of 0.9 ps for the S_2_ state in terms of the global fitting.

Although there also have been absent of the experimental evidence regarding the lifetime of S_2_ state for nitroaniline so far, previous TRPES (time-resolved photoelectron imaging) works on the benzene derivatives^44^,^45^,^46^,^47^ such as benzene, toluene, indene, styrene, o-xylene, and phenylacetylene, indicated that the internal conversions from S_2_ state to S_1_ one are commonly ultrafast within 100 fs, much shorter than 0.9 ps derived from our work. However, some experiments on the aromatic hydrocarbon benzophenone with excitation at 266 nm reported that the lifetime of the S_2_ → S_1_ transition is between 0.1–0.2 ps in gas phase^40^ and 0.53 ps in acetonitrile solvent^37^. In addition, 0.59 ps lifetime of S_2_ → S_1_ was reported for a benzophenone derivative (p-iodobenzophenone) in acetonitrile media^37^. Longer relaxation time with 0.8 ps lifetime was observed from the femto-TA transient absorption spectrum of HNS for its S_2_ → S_1_ decay in acetontrile media^28^. The *ab initio* MD study on the uracil in solution^42^, which exhibited good consistency with the experimental observations, also found that 50% of the excited trajectories are still trapped in S_2_ after 1 ps, indicating that the depopulation of the S_2_ state slowly occur due to internal conversion to the S_1_. Recently, longer relaxation time within a few picoseconds from the S_2_ state to the S_1_ one was reported by Crespo-Hernández, ^4^ who used femto-TA experiment in combination with quantum chemistry calculation (CASSF and ab initio surface-hopping simulation) to study the excited-state dynamics of the purine free base and 9-methylpurine. Their results indicated that the excitation of these purine derivatives in aqueous solution at 266 nm results primarily in fast conversion of the S_2_ (π, π^*^) state to the vibrational excited S_1_ (n, π^*^) state within a few picoseconds and then an intersystem crossing occur within hundreds of picoseconds. Thus, combined with our work, it can be drawn that the S_2_ → S_1_ internal conversion would take place in the time range between a few femtosecond and a few picosecond, depending on the structures of various molecules and the environments used.

#### S_1_ state

The spectrum of the second species with a sharp negative signal peaked at 660 nm is assigned to the S_1_ state and its lifetime is estimated to be 6.4 ps by the global fitting, similar to results from 4- nitrated polyaromatic, 2-nitrated polyaromatic and 11-nitrated polyaromatic, S_1_ lifetimes of which were reported to be 4.4 ps, 10 ps and 3 ps, respectively^21^. Due to the vital role of the S_1_ decay in controlling the performance of the photochemical and photobiological systems, previous works in the fields mainly focused on the subject, in particular for the intersystem crossing (ISC).

As revealed, there are usually rapid S_1_ decays for most nitroaromatics as short as a few hundred femtoseconds due to a strong coupling of the S_1_ state with T_1_ state^23^,^24^,^26^,^48^. For example, the femtosecond-resolved experiments on 2-nitrofluorene shows ultrafast intersystem crossing which depopulates the S_1_ emissive state within less than a picosecond. However, introduction of NH_2_ to 2-nitrofluorene leads to a much longer lifetime of 100 ps in non-polar cyclohexane solvents^48^. In addition, S_1_ decays of the singly nitrated pyrenes (NP) was reported to be strongly dependent on the position of NO_2_, leading to that the decay of the fluorescence states can be of several hundred picoseconds or even enter the nanosecond scale in acetonitrile^23^,^49^,^50^. For example, S_1_ in 1-nitropyrene is short-lived (up to 3 ps) while, due to the lack of an appropriate receiver triplet state, S_1_ states of 4-nitropyrene and 2-nitropyrene have 0.41 ns and 1.2 ns lifetimes, respectively^23^. For 3-nitropyrene, it took place on a 100 ps time scale^50^.

#### Intermediate state S* in the intersystem crossing

Since the fluorescence emission is absent for ONA, there should exist a significant nonradioactive decay pathway in its S_1_ excited state. Thus, we focused on its ISC pathway. As revealed, the intersystem crossing channels would become highly efficient when the singlet and upper triplet states have similar energies and appropriate electronic configurations^51^,^52^,^53^, while ISC would be suppressed by symmetry when the participating singlet and triplet states are composed of essentially same orbitals^54^ in terms of EI-Sayed rules^55^.

**Figure 7:**
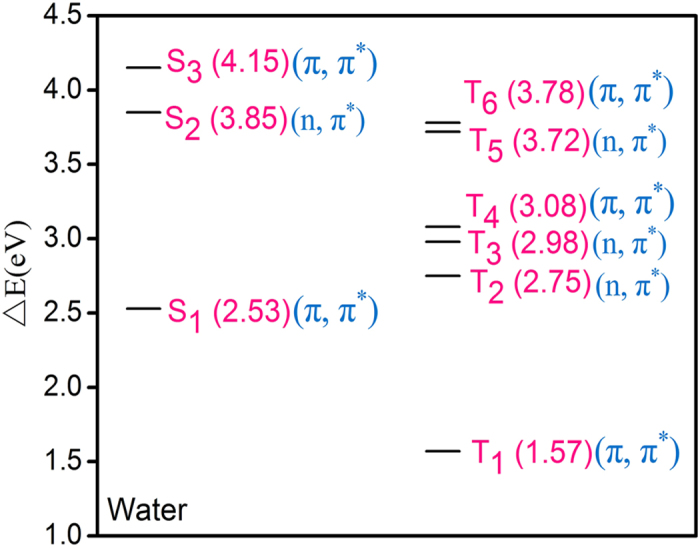
The excitation energies with respect to the electronic ground state are obtained using TDDFT calculation at the level of B3LYP/6-311++G(d,p)/PCM, based on the optimized geometries of the first singlet excited state and the first triplet excited one, respectively.

Thereby, we analyzed the energy levels and electronic nature for some low-lying single and triple excited states of ONA (see Fig. 7) through TD-DFT calculations at the level of B3LYP/6-31++ G (d, p), based on the optimized S_1_ and T_1_ structures. The calculation result indicates that the two optimized structures have same (π, π*) characters. In addition, the energy gap between the S_1_ state and the T_1_ one is also significant (ΔE = 0.96 eV). As a result, the direct transition from S_1_ (π, π^*^) to T_1_ (π, π^*^) should be prohibited according to EI-Sayed rules. Then, it is reasonable to assume that the occurrence of ISC transition should be mediated by one intermediate state for the ONA. The assumption is also supported by the observation from Fig. 3 that a sharp peak abruptly appear at near 675 nm at the 20 ps time delay. Consequently, the third spectrum can be assigned to be the intermediate state (termed as S^*^) and its lifetime is estimated to be 19.6 ps by the global fitting analysis, close to 20 ps shown in Fig. 3.

The intermediate states in the ISC process were previously reported for some systems. For example, Aloise^56^ proposed an intermediate state (termed as IS) existed in the ISC process for benzophenone through the pump-probe subpicosecond absorption experiments in combination with multivariate curve resolution, in which the transition lifetimes of S_1_ → IS and IS → T_1_ were estimated to be ~6.5 ps and 10 ps, respectively. The intermediate states S^*^ were observed for the ISC processes of carotenoid^39^, spheroidene^57^ and 1-nitronaphthalene^53^ lifetimes of which were estimated to be 30 ps, 5.5 ps and the time scales of 1–16 ps, respectively. In addition, previous works on some nitroaromatics^24^,^33^,^58^,^59^ and purine nucleobase derivatives^4^ further proposed that the indirect ISC pathway from S_1_ to T_1_ states can be performed through one particular upper triplet states. The specific upper triplet state has isoenergetic energy and appropriate spin–orbit coupling character, leading to ultrafast ISC times as short as a few hundred femtoseconds. Whereas for some nitroaromatics, there are lack of the appropriate receiver triplet state upper the T_1_ energy. Consequently, their ISCs occur on a much longer time scale of hundreds of picosecond, even up to nanosecond^46^.

As shown in Fig. 7, no any triplet state is placed below the S_1_ state energy level with exception of T_1_ for ONA, implying that there is no an appropriate “receiver” triplet state required for the fast ISC process. However, different from the time scale within femtosecond and nanosecond of S_1_ lifetime reported, the S_1_ state of ONA has the lifetime of 6.4 ps, derived from the MCR-ALS and the global fit analysis. Thus, it is reasonable to conjecture that the intermediate mediating the ISC pathway of ONA is different from the character of the upper triplet state, maybe mixed with the singlet state character and the triplet one. Further aspects of addressing the nature of intermediate state are beyond the work.

#### T_1_ state

The fourth spectrum should be assigned to be T_1_ state. A detail comparison of T_1_ time-dependent concentration with that of S^*^ state clearly shows that there is an opposite trend between the two states (vide Fig. 5). Namely, depopulation of S^*^ corresponds to repopulation of T_1_ state, similar to the population relationship between S_1_ state and S^*^ one. The lifetime of T_1_ is estimated to be 2 ns by the global fitting analysis, significantly exceeding the experimental time range of 400 ps relaxation. The conclusion is consistent with the finding above that the persistence of the positive absorption at long wavelengths still exists even at 400 ps delay time (vide Fig. 2), which imply the formation of a new long-lived state. It was reported that the absorption of the triplet state of the benzophenone^56^,^57^, HNS^28^ and 9-methylpurine^4^ could persist up to the maximum delay of 1.6 ns, ~4 ns and >3 ns time scale, respectively, which are in line with the nanosecond time scale of the triplet state derived from our work.

## Conclusion

By introducing advanced chemometric method (MCR-ALS) and quantum mechanics calculation, we proposed a joint strategy to resolve the femtosecond transient absorption for ONA. The results revealed that the ultraviolet excitation populates primarily the S_3_ (π, π^*^) state in the Franck–Condon region. The internal conversion from S_3_ to S_2_ should be too ultrafast to be identified from our femtosecond spectrum with 200 fs resolution time. However, the decay from S_2_ state to S_1_ one was determined to be within 0.9 picosecond. It is noteworthy that one intermediate was detected in the ISC pathway from the S_1_ state to T_1_ one and the existence of the intermediate results in fast decay of S_1_ → S^*^ within 6.4 ps, which should contributed to the absence of fluorescence for ONA. The 6.4 ps lifetime of S_1_ state is different from some previous observations concerning ultrafast S_1_ deactivation within femtosecond time scale resulted from the existence of appropriate upper triplet state strong coupling with S_1_, and also distinguished from ultraslow decay on the nanosecond time scale due to the absence of appropriate upper receiver triplet state in the ISC pathways reported for some nitroaromatics. The decay time from the intermediate S^*^ state to the T_1_ one was estimated to be 19.6 ps. The following T_1_ was populated and its lifetime was estimated to be up to be 2 ns. Based on the observations, it can be assumed that relatively long-lived S^*^ and very long-lived T_1_ states, in particular for T_1_ state, should play a dominant role as precursors to various nitroaromatic and explosive photoproducts because they cannot directly compete with the other possible deactivation process with shorter or similar lifetimes.

To the best of our knowledge, it is for the first time to combine the advanced chemometrics method (MCR-ALS) with quantum chemistry calculation to explore an efficient and facile way to thoroughly resolve the femtosecond transient adsorption. More importance, some novel observations were obtained from ONA and extend knowledge’s regarding the photophysics of the nitroaromatics, which in turn is helpful for the understanding of photoinduced reactive channels of nitroaromatics and the initial ignition process of explosives like TATB, DATB and TNA.

## Experimental Method

### Transient Absorption Measurements

The typical transient absorption experimental setup were described in detail before^60^,^61^. Briefly, a regenerative amplified laser system was used to generate fundamental pulse of duration of 35 fs centered at 800 nm, with an energy per pulse of 1 mJ and repetition rate of 1 kHz. Another harmonics fundamental pulse centered at 400 nm was obtained by a 1 mm thick BBO crystal. The 266 nm pump beam attenuated to ~5 μJ as excitation pulse was obtained by sum frequency mixing of the first and the second pulses. The white-light-continuum pulses obtained from CaF_2_ crystal were spilt into the experimental probe beam with the range of 400–710 nm and the reference one by a beam-splitter. The probe pulse passed through an optical delay line and overlapped with the pump in sample cell at an intersection angle of 5˚. And the polarization angle between pump and probe beams was set at a magic angle of 54.7˚. The fundamental, pump and the probe pulses were controlled by three optical shutters. The probe and the reference pulses were detected by a two dimensional CCD detector (PI-MAX, 1024 × 256 pixel array) equipped with a grating inside spectrometer (Princeton, SpectraPro 2500i). The probe and the reference image signals from CCD were sent to computer by a 16-bit analog-to-digital converter (ADC). The experiment control and data acquisition were performed using a labview program. By means of averaging approximately 1600 pulses, the noise level lower than 2 mOD could be achieved. The instrumental response of the system was determined to be ~200 fs by cross-section measurement between pump and probe pulses, which was also used to measure the precise zero time-delay at each probe wavelength. The chirp of white-light-continuum pulse was measured at the position of the sample cell by the use of the optical Kerr effect of solvent and was used to correct the dispersion of relative delay time in the time-resolved data. The 0.7 mmol/L of ONA in water was prepared under dark room and circulated flow by a device to avoid re-excitation. The cross-correlation function was also used to determine the precise zero time-delay at each probe wavelength.

### Multivariate Curve Resolution-Alternating Least Squares (MCR-ALS)

In general, the experimental matrix ***D*** of femtosecond-TA can be described by a bilinear model given in Eq. (1)


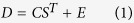


Where the matrix ***C*** (*m *×* k*) contains the time dependent concentration profiles of *k* pure contribution at *m* delays and matrix ***S***^***T***^ (*k *×* n*) contains the corresponding transient spectra at *n* wavelengths. The error matrix ***E** (m × n)* contains the residuals not explained by the *k* components. Multivariate curve resolution-alternating least squares (MCR-ALS)^17^,^62^ was applied to resolve Eq.(1) in order to select the real contributions of the pure components represented by the concentration profiles ***C*** and spectra profiles ***S***^***T***^ from the mixed nonselective information. The first step of MCR-ALS algorithm is to estimate the appropriate number of components composing the experimental matrix ***D***, which is usually obtained by singular-value decomposition (SVD)^63^,^64^. However, the SVD alone cannot settle correctly threshold that separates chemical contributions from noise. Thus, instead of SVD analysis on the full data matrices, evolving factor analysis (EFA)^65^,^66^ was used to perform successive SVD analysis on the gradually increasing submatrics by means of adding a row at a time from the top of the matrix to the bottom (forward EFA). The emergence and evolution of the singular values can be followed individually with increasing time. Also, EFA from bottom to top (backward EFA) was carried out using the same approach, in which the emergence and evolution of the singular values can be followed in reverse sequence and their disappearance can be observed with the increasing time. Taken the forward EFA result together with the backward EFA one, estimation of the profiles ***C*** and ***S***^***T***^ can be obtained and input as initial solution in the MCR-ALS procedure. The ALS algorithm is used to fit iteratively the ***C*** and ***S***^***T***^ matrices to the experimental matrix ***D***. At each iterative cycle of the optimization, matrices ***C*** and ***S***^***T***^ are calculated under constraints minimizing the reproduction error ***E***. Non-negativity and unimodality constraints for concentration profiles were applied during the ALS optimization order to get meaningful results^41^,^56^. The lack of fit (Eq. 2) can be used as global estimator of the fitting error of the transient spectra data.


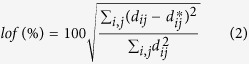


where *d*_*ij*_ is an element of the experimental data matrix and (*d*_*ij*_*–d*_ij_^*^)^2^ is the related residual value obtained from the difference between the experimental matrix ***D*** and the reproduced matrix***CS***^***T***^ derived by MCR-ALS.

### Quantum Mechanics Calculation

The equilibrium geometries of the ground state S_0_ and the triplet excited state T_1_ were optimized using density function theory (DFT)^67^ method at the level of B3LYP^68^/6-311++ G(d, p) while the optimization of the S_1_ state was carried out using time-dependent density functional theory (TDDFT)^69^ at the same level. The vibrational frequency calculations confirm that the optimized structures are true minimum on the potential energy surfaces. Based on the optimized geometries, TD-DFT calculations were carried out to obtain the transition properties at the level of B3LYP/6-311++ G (d, p). All calculations above included the water environment using the polarizable continuum model (PCM)^70^,^71^. The Gaussian 09 suite of programs^72^ was used for all calculations.

## Additional Information

**How to cite this article**: Yi, J. *et al.* A Combination of Chemometrics and Quantum Mechanics Methods Applied to Analysis of Femtosecond Transient Absorption Spectrum of Ortho-Nitroaniline. *Sci. Rep.* **6**, 19364; doi: 10.1038/srep19364 (2016).

## Figures and Tables

**Table 1:** Vertical excitation energies in eV (nm), oscillator strengths and transition nature of the low-lying singlet excited states of the ONA in water, calculated by TDDFT method at the level of B3LYP/6-311++ G(d, p) with the framework of PCM.

Electronic transition	Transition energy/eV(nm)	Oscillator strength	Transition character
S_0_ → S_1_	3.06(405)	0.12	π → π^*^
S_0_ → S_2_	3.85(322)	0.00	n → π^*^
S_0_ → S_3_	4.38(283)	0.22	π → π^*^
S_0_ → S_4_	4.53(273)	0.00	n → π^*^
S_0_ → S_5_	5.12(242)	0.02	π → π^*^
S_0_ → S_6_	5.45(227)	0.01	π → π^*^
